# Growth hormone axis in chronic kidney disease

**DOI:** 10.1007/s00467-007-0527-x

**Published:** 2008-01-01

**Authors:** Shefali Mahesh, Frederick Kaskel

**Affiliations:** 1 Albert Einstein College of Medicine, Children’s Hospital at Montefiore, Bronx, NY 10467 USA; 2 Division of Pediatric Nephrology, Children’s Hospital at Montefiore, 111 East 210th street, Bronx, NY 10467 USA

**Keywords:** Growth hormone, Insulin-like growth factor, Chronic kidney failure, Growth failure, Treatment

## Abstract

Chronic kidney disease (CKD) in children is associated with dramatic changes in the growth hormone (GH) and insulin-like growth factor (IGF-1) axis, resulting in growth retardation. Moderate-to-severe growth retardation in CKD is associated with increased morbidity and mortality. Renal failure is a state of GH resistance and not GH deficiency. Some mechanisms of GH resistance are: reduced density of GH receptors in target organs, impaired GH-activated post-receptor Janus kinase/signal transducer and activator of transcription (JAK/STAT) signaling, and reduced levels of free IGF-1 due to increased inhibitory IGF-binding proteins (IGFBPs). Treatment with recombinant human growth hormone (rhGH) has been proven to be safe and efficacious in children with CKD. Even though rhGH has been shown to improve catch-up growth and to allow the child to achieve normal adult height, the final adult height is still significantly below the genetic target. Growth retardation may persist after renal transplantation due to multiple factors, such as steroid use, decreased renal function and an abnormal GH–IGF1 axis. Those below age 6 years are the ones to benefit most from transplantation in demonstrating acceleration in linear growth. Newer treatment modalities targeting the GH resistance with recombinant human IGF-1 (rhIGF-1), recombinant human IGFBP3 (rhIGFBP3) and IGFBP displacers are under investigation and may prove to be more effective in treating growth failure in CKD.

## Learning objectives:

1. To review the normal growth pattern in childhood and its alteration in chronic kidney disease.
2. To understand the mechanisms believed to be responsible for growth failure in chronic kidney disease.
3. To understand the new evidence supporting therapy for growth failure in chronic kidney disease.
4. To identify targets for future therapies for treatment of growth retardation in chronic kidney disease.

## Introduction

Growth retardation is a common result of chronic kidney disease (CKD) in childhood. Children with CKD fail to achieve the final adult height consistent with their genetic potential [1]. Data from the North American Pediatric Renal Trials and Collaborative Studies (NAPRTCS) 2005 database revealed that 36.9% of children with CKD had statutory growth impairment. Even though the growth failure was correlated with the degree of renal impairment, those with mild reduction of glomerular filtration rate (GFR) also exhibited short stature. Children were short at initiation of dialysis, with younger age associated with more severe growth failure and little improvement in height standard deviation score (SDS). The mean height deficit for all renal failure patients was −1.85 (less than the third percentile of their healthy peers) at transplantation. This deficit was greater for male patients and for younger subjects. Those below age 6 years benefited most from transplantation in demonstrating acceleration in linear growth. For subjects aged 6–12 years, the linear growth remained stable, and those older than 12 years of age had no increase in their height deficit score [2–4].

Despite advances in medical care, growth failure in CKD is associated with increased morbidity and mortality. Furthet al. demonstrated from the United States Renal Data System (USRDS) database that patients with severe-to-moderate growth failure had increased hospitalization rates and increased risk of death [5].

Growth retardation is assessed by the SDS or height deficit score, which measures the patient’s height in comparison with that of unaffected children of similar age. Wong et al. demonstrated that, among pediatric patients on dialysis or after transplantation, each SDS decrease in height was associated with an increase in death by 14% [6].

## Alteration of normal growth patterns in CKD

Linear growth is unique to childhood. Complex biological processes are responsible for maintaining normal growth.

Infancy is the fastest growing period of childhood; one-third of the total growth occurs in the child’s first 2 years of life, and this is mainly nutrient dependant. This growth is markedly decreased in congenital CKD, with the greatest height deficit occurring in the child’s first year of life, especially during the first 4 months [7]. Thus, the earlier the onset of kidney disease, the more severe the growth disturbance.

Additional factors affecting growth are inadequate protein and calorie intake, water and electrolyte losses in polyuric and salt wasting conditions, anemia, metabolic acidosis, renal osteodystrophy, and resistance to hormones mediating growth. Early nutritional intervention and prevention of metabolic deficits of renal failure can preclude the development of growth failure in this period. Conservative treatment (no use of growth hormone) in pre-dialysis patients from birth to 3 years, revealed a favorable growth rate, with height velocity at 22.2 cm/year, 10.9 cm/year and 7.6 cm/year for each year, respectively, all of which were higher than the lower two standard deviation scores [5]. Further discussion of these factors is beyond the scope of this article.

During the mid-childhood period, a constant growth rate of 5–7 cm/year is demonstrated, mainly under the control of growth hormone (GH) and thyroid hormone. Children with congenital CKD grow at the percentile achieved at the end of 2 years of life. Even though one of the most important predictors of growth impairment is the degree of renal insufficiency, significant short stature has been seen at all levels of renal function [3, 7].

At the onset of puberty, the growth hormone/insulin-like growth factor (GH/IGF-1) axis is activated by small increases in estrogen and testosterone in girls and boys, respectively. The onset of puberty is delayed in adolescents with CKD; peak height velocity is delayed by approximately 2.5 years. The pubertal growth spurt is delayed, shortened and associated with a reduced growth velocity. The pubertal height gain is about 65% of that seen in healthy children without CKD and is likely due to the shortened growth spurt [1]. These effects of delayed puberty in CKD are mediated by a loss of the normal pulsatile hypothalamic release of the gonadotropin-releasing hormone (GnRH).

## GH and IGF axis

**Fig. 1:**
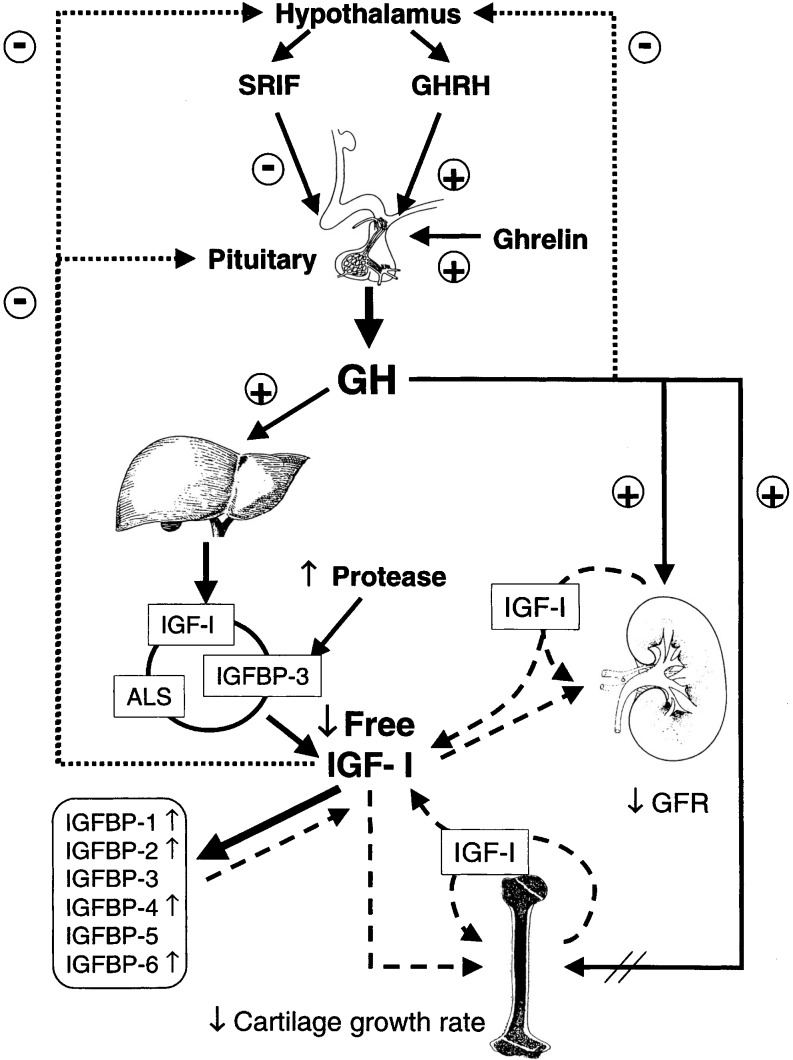
GH/IGF-1 axis in CKD: deranged somatotropic axis in chronic renal failure. The GH/IGF-I axis in CRF is changed markedly, compared with the normal axis shown here. In CRF the total concentrations of the hormones in the GH/IGF-I axis are not reduced, but there is reduced effectiveness of endogenous GH and IGF-I, which probably plays a major role in reducing linear bone growth. The reduced effectiveness of endogenous IGF-I likely is due to decreased levels of free, bioactive IGF-I as levels of circulating inhibitory IGF-binding proteins (*IGFBPs*) are increased. In addition, less IGF-I is circulating in the complex with acid labile subunit (ALS) and IGFBP-3 as a result of increased proteolysis of IGFBP-3. Together, these lead to decreased IGF-I receptor activation and a decreased feedback to the hypothalamus and pituitary. Low free IGF-I and high IGFBP-1 and IGFBP-2 levels probably contribute to reduced renal function and lead to reduced stature. The direct effects of GH on bone, which are poorly understood, also are blunted. Reprinted from [8] with permission

Growth hormone is the key endocrine regulator of postnatal growth (Fig. 1) [8]. The anterior pituitary secretes growth hormone in a pulsatile manner stimulated by the growth hormone releasing hormone (GHRH) and inhibited by somatostatin. GH mediates its somatotropic actions directly and mainly through IGF-1. Ghrelin, a growth hormone-releasing peptide expressed in the stomach and hypothalamus, is involved in hormonal and nutritional regulation of GH release [9].

Growth hormone and the IGF-1 axis play a major role in growth failure in CKD. Random fasting serum levels of GH are normal or increased in children and adults with CKD, depending on the extent of renal failure. The half-life of GH is prolonged, due to decreased metabolic clearance secondary to decreased functional renal mass in proportion to the degree of renal dysfunction. A high normal calculated GH secretion rate and amplified number of GH secretory bursts have been reported in pre-pubertal children with end-stage renal disease, likely due to attenuated IGF-1 feedback [8]. This has led to the concept of GH insensitivity or resistance in uremia.

Pubertal patients with advanced CKD have a reduced GH secretion rate, indicating altered sensitivity of somatotropic hormones to the stimulatory effects of sex steroids [10].

## GH resistance

### GH receptors

One mechanism for GH resistance is a reduced density of GH receptors in target organs. Determination of the concentration of serum growth hormone binding protein (GHBP), which is a cleaved product of the GH receptor, may be used to assess GH receptor density in tissues, particularly liver, since GHBP is derived mainly, but not exclusively, from the liver. GHBP is low in children and adults with CKD and proportionate to the degree of renal dysfunction. Serum GHBP correlates with both spontaneous growth rate and response to GH therapy, and it is an indirect indicator of sensitivity to both exogenous and endogenous GH [11, 12]. However, there is controversy as to the reliability of serum GHBP level as a marker of GH receptor levels in specific tissues [13].

### Janus kinase/signal transducer and activator of transcription signaling

**Fig. 2:**
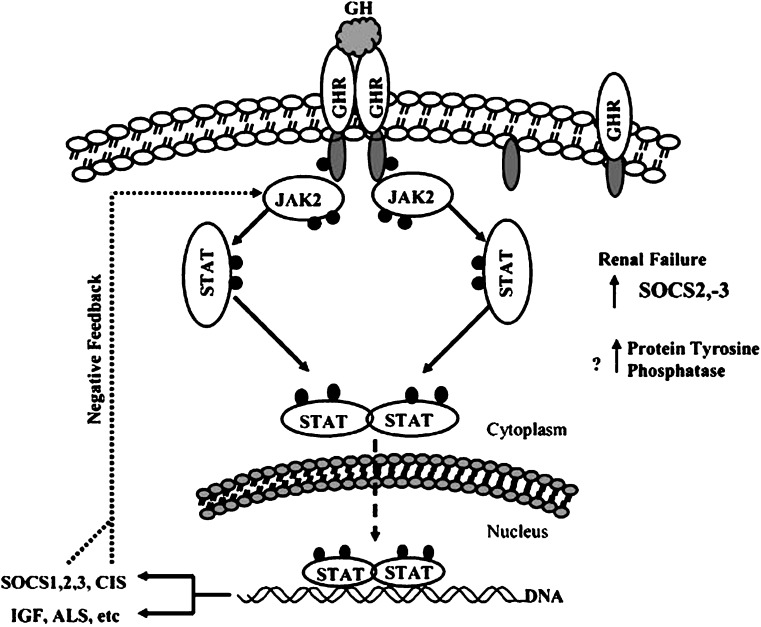
Growth hormone-mediated JAK/STAT signal transduction. GH activates several signaling pathways via JAK2, including the JAK/STAT pathway [22, 23]. Binding of GH to its receptor (GHR) activates JAK2, which then self-phosphorylates. This is followed by phosphorylation of the GHR and, subsequently, STAT 1a, STAT 3, STAT 5a, and STAT 5b, members of a larger family of cytoplasmic transcription factors. These phosphorylated STATs form dimers that enter the nucleus, where they bind to specific DNA sequences and activate their target genes, IGF-1 and some suppressors of cytokine signaling (*SOCS*). Deletion of STAT5 expression leads to retarded body growth, and STAT5b is required for GH-mediated IGF-1 gene expression. In renal failure phosphorylation of JAK2 and the downstream signaling molecules STAT5, STAT3, and STAT1 is impaired, as are the nuclear levels of phosphorylated STAT proteins. This important cause of uremic GH resistance may result, in part, from up-regulation of SOCS2 and SOCS3 expression with suppressed GH signaling and also from increased protein tyrosine phosphatase activity, with enhanced dephosphorylation and deactivation of the signaling proteins. Reprinted from [14] with permission

Another mechanism for GH resistance in uremia is a defect in post-receptor GH-activated Janus kinase/signal transducer and activator of transcription (JAK/STAT) signaling (Fig. 2) [14]. GH action is mediated by the binding of GH to the growth hormone receptor (GHR), resulting in its dimerization and the auto-phosphorylation of the tyrosine kinases, Janus kinase 2 (JAK2), which, in turn, stimulates phosphorylation of signaling proteins, STAT proteins STAT1, STAT3 and STAT5. Upon activation, these STAT proteins translocate to the nucleus and activate GH-regulated genes. An intact JAK2-STAT5b signaling pathway is essential for GH stimulation of IGF-1 gene expression. In uremia, a defect in the post-receptor GH-activated JAK2 signal transducer and STAT transduction is described as one of the mechanisms of GH resistance [14].

The JAK2/STAT pathway is regulated, among other factors, by suppressor of cytokine signaling (SOCS) proteins, which is induced by GH. These proteins bind to JAK2 and inhibit STAT phosphorylation [15]. Up-regulation of SOCS has been described in inflammatory states and may play a similar role in CKD [16].

### IGF and IGFBP

Daughaday et al. proposed the somatomedin hypothesis: GH leads to an increase in IGF-1 (formerly called somatomedin) c) from the liver, which, in turn, reaches the growth plate to mediate its action [17]. However, it was later demonstrated that all the effects of GH are not mediated through IGF-1. Also, the liver is not the only source of IGF-1, and it is produced in other tissues. Circulating IGF-1 mainly derived from the liver acts as an endocrine hormone. IGF-1 produced locally acts as a paracrine/autocrine hormone. The majority of circulating IGF in the serum exists as a 150 kDa complex, including the IGF molecule, IGF binding protein 3 (IGFBP3) and the acid labile subunit (ALS). IGF-1 gene knockout in mice results in miniature mice that die soon after birth. Knock-out of IGF-1 in the liver alone results in relatively normal sized mice with a 75% reduction in circulating IGF-1 levels, suggesting that local IGF-1 may be more important for body growth than liver IGF-1 [18]. ALS knock-out mice also exhibit relatively normal growth and development, despite a 65% reduction in circulating IGF-1 levels. Disruption of both liver IGF-1 and ALS genes results in 85–90% reduction in circulating IGF-1 levels and significant reduction in linear growth, suggesting that a threshold concentration of IGF-1 is necessary for normal growth [19].

IGF-1 is the predominant IGF during the rapid growth period of puberty. The levels of IGFs (IGF-1 and IGF-2) are normal in pre-terminal renal failure. In end-stage renal disease IGF-1 level is normal or decreased and levels of IGF binding proteins (IGFBP) are increased, thus resulting in a net decrease in IGF bioactivity. Free IGF-1 is decreased in proportion to renal failure [20, 21].

IGF-1 is an anabolic hormone; it binds to the IGF-1 receptor (IGF-1R) at the α subunit, where it activates tyrosine kinase intrinsic to the IGF-1Rβ subunit. In experiments with rats with CKD, skeletal muscles show decreased serum levels of IGF-1 and IGF-1 mRNA, while IGF-1R mRNA and IGF-1R numbers are increased, with normal binding affinity. However, there is resistance to post-receptor signal transduction; both auto-phosphorylation of the IGF-1R tyrosine kinase and activity of the IGF-1R tyrosine kinase to the exogenous insulin receptor substrate-1 (IRS-1), a natural substrate for IGF-1 receptor tyrosine kinase, are diminished in skeletal muscle of CKD rats. These abnormalities may account for the resistance of IGF-1 to protein turnover in skeletal muscles in CKD [22].

IGFs are transported in plasma bound to IGF binding proteins (IGFBPs), which are responsible for extending their half-life and controlling their bioavailability. There are six different IGFBPs, based on their respective amino acid sequence. Of the IGFs in the serum, 75% circulate as a ternary complex bound to IGFBP3 and another subunit, acid labile subunit (ALS), to form major 150 kDa complexes. This acts as a reservoir and cannot cross the endothelial barrier. Some 20–25% of IGFs are present as smaller binary complexes with other IGFBPs forming the minor complex (35 kDa) [23]. Approximately 97% of IGF is bound; 1% of IGF-1 is in a free bioactive form. In healthy children there is a 25% excess of IGFBPs over IGF, while, in pre-terminal renal failure, IGFBPs are 150% in excess of IGFs, and in end-stage renal disease they are 200% in excess, thus reducing the bioavailability of IGF [21].

IGFBP3 is the most abundant of all the binding proteins. IGFBP3 and IGFBP5 are similar in many aspects; structurally, they are both closely related, both potentiate IGF action and are up-regulated by GH. The serum level of intact IGFBP3 is normal in CKD, while the fragmented fraction (29 kDa) is increased but has a reduced affinity for peptides [24]. IGFBP5 level is not altered, but it exists mostly in the fragmented form.

Intact IGFBP1, IGFBP2, IGFBP4 and IGFBP6 are elevated in CKD in relation to the degree of renal dysfunction [25]. IGFBP1, IGFBP2 and IGFBP6 are growth inhibitors of IGF-dependant proliferation of chondrocytes. Increased levels of IGFBP1 and IGFBP2 have been shown to correlate negatively with height [26]. Ulinski et al. analyzed serum levels of IGFBP4 and IGFBP5 in 89 CKD patients. There was a fourfold increase in IGFBP4, while IGFBP5 was not significantly increased. IGFBP4 level was inversely correlated with glomerular filtration rate and standard height. IGFBP5 level was positively correlated with standard height [25].

## Ghrelin

Ghrelin is a newly discovered, 28-amino acid peptide, with growth hormone releasing properties [growth hormone secretagogue (GHS)], expressed most abundantly in the stomach and also in smaller quantities by pancreatic islet cells and hypothalamus. Ghrelin levels are increased by fasting, cholinergic stimulation, estrogen and recombinant hIGF-1, and are decreased by food intake [27].

Ghrelin has been shown to increase the release of GH which is amplified by co-administration of GHRH, and acts at the level of the hypothalamus and pituitary. The role of ghrelin in the growth abnormalities in CKD has yet to be defined.

## Treatment with recombinant human growth hormone

**Fig. 3:**
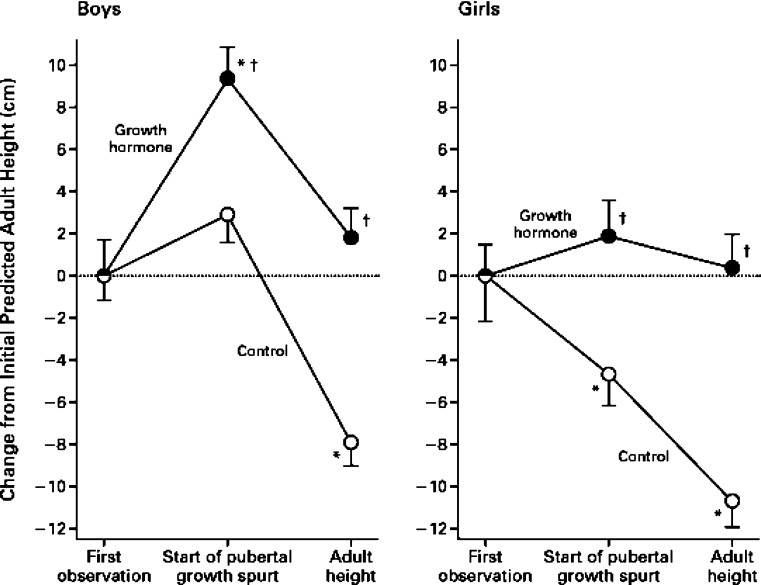
Growth hormone treatment in chronic kidney disease: change from initially predicted adult height at baseline in 38 children (32 boys and six girls) with chronic renal failure who received growth hormone treatment compared with 50 control children with chronic renal failure who did not receive growth hormone, according to gender. Values are means ± SD. *Asterisks* indicate significant differences from the previous period (*P* < 0.001) and *daggers* indicate significant differences from the children who were not treated with growth hormone (*P* < 0.001). Reprinted from [1] with permission

Studies in the past have shown that therapy with recombinant human growth hormone (rhGH) in CKD is safe and efficacious, and that it increases growth rate and improves standardized height [28, 29]. Haffner et al. studied the effect of growth hormone treatment on final adult height of children with CKD (Fig. 3) [1]. In contrast to the controls that had persistent growth failure, children treated with rhGH demonstrated sustained catch-up growth. In treated children the standardized height increased from baseline to a mean final adult height of 1.6 ± 1.2 SD below normal, while, in untreated control children, it decreased from baseline to a mean final adult height of 2.1 ± 1.2 SD below normal. Of the children treated with rhGH, 65% reached a final adult height within the normal range, although it was significantly below the genetic target [1].

Poor growth outcomes after renal transplantation are associated with corticosteroid use, persistent CKD and abnormalities in the GH-IGF-1 axis. The use of rhGH after transplantation leads to catch-up growth associated with increase in IGF-1 levels [26]. Using the NAPRTCS database of 513 renal transplant recipients receiving rhGH, Fine and Stablein demonstrated that final adult height was superior in rhGH treated patients compared to controls. Allograft function and graft failure rates were similar in both groups, with no increase in adverse events [30].

RhGH treatment in renal failure is associated with an elevation in serum free IGF-1 [31]. RhGH increases IGFBP3, IGFBP4, and IGFBP5, decreases IGFBP1 and has no effect on IGFBP2 and IGFBP6. RhGH also increases IGFBP4, but does not correlate with change in height SDS, since most of the IGFBP4 in serum is fragmented [25, 31]. RhGH-mediated increase in IGFBP3, IGFBP5 and ALS results in increased ternary complexes and correlates positively with the increment in height SDS [31].

Among the growth-promoting effects of treatment with rhGH in CKD are anabolic effects, demonstrated by increase in body weight and mid-arm muscle circumference. The recommended dose approved for treatment of growth failure in CKD is 0.35 mg/kg per week (28 IU/m^2^ per week) [3].

## Targets for future therapy:

### Treatment with recombinant IGF-1

The GH resistance associated with CKD may be amenable to treatment with recombinant human IGF-1 (rhIGF-1). In children with GH-receptor deficiency or GH-inactivating antibodies, rhIGF-1 treatment resulted in a modest increase in growth velocity and height SDS, although less than that expected with rhGH [32].

In addition, short-term administration of rhIGF-1 has been shown to increase glomerular filtration rate and renal plasma flow in patients with end-stage renal disease and in healthy subjects. Vijayan et al. demonstrated sustained improvement in renal function in a 31-day randomized, double-blinded, placebo-controlled trial of 15 patients with advanced CKD, who had received intermittent doses of rhIGF-1 [33].

One reason for the use of IGF-1 treatment in CKD is that while patients are GH sufficient, they are GH resistant. Therefore, rhIGF-1 may be more beneficial than GH as therapy in CKD and merits further investigation as an agent for treatment of growth failure and improvement in renal function in CKD [34].

### Combined use of rhGH and rhIGF-1

Animal studies have shown that the combined use of rhGH and rhIGF-1 has an independent and additive effect. While whole-body growth in uremic rats was similar with treatment with either agent, an additive effect on longitudinal growth and anabolism was demonstrated when both agents were administered together [35].

### Combined use of rhIGF-1 and rhIGFBP3

One of the concerns with the use of rhIGF-1 in children with normal GH production is that rhIGF-1 may suppress endogenous GH, IGFBP3 and ALS production, which may have a detrimental effect on growth. The other adverse effect of the use of rhIGF-1 observed in GH receptor-deficient patients is hypoglycemia, due to low levels of IGFBP3 and increased level of free IGF-1 available for binding to insulin receptors [32]. Both these effects can be reduced by the combined use of rhIGF-1 and rhIGFBP3 [36].

### IGFBP displacers

At least two different IGF-1 analogs have been made, which have an affinity for IGFBPs but have no effect on IGF receptors, thus “displacing” IGF-1 from IGFBPs and elevating free IGF-1 levels. Animal studies in hypophysectomized rats and dwarf dw/dw rats treated with IGFBP displacers demonstrated increased kidney size, improved renal function, and the stimulation of weight gain and bone growth. This effect was enhanced when treatment with IGFBP displacers was combined with rhIGF-1 [37].

## Conclusion

This review of the literature lends support to the concept that CKD is associated with GH “resistance”. Despite adequate treatment with rhGH and improvement in catch-up growth, children with CKD display a final adult height that is often below the genetic target. The potential for newer therapies with rhIGF-1, combined use of rhGH and rhIGF-1, combined use of rhIGF-1 and rhIGFBP3 or IGFBP displacers to improve both the short and long term outcomes in the treatment of the disturbances in the GH/IGF-1 axis in CKD awaits future investigations.

## Questions (answers appear after the reference list)

1. All the factors mentioned below are causes of growth failure in chronic kidney disease except
  - increased sensitivity to hormones mediating growth
  - anemia
  - metabolic acidosis
  - inadequate caloric intake
  - renal osteodystrophy.
2. What are the mechanisms of growth hormone resistance in uremia?
  - increased GH receptor density in target organs
  - defect in post-receptor JAK/STAT signaling
  - growth hormone deficiency
  - increased IGFBPs and decreased free IGF-1 levels
3. How does treatment with growth hormone modify the altered GH-IGF1 axis?
  - increased GH levels in serum
  - increased IGF-1 levels
  - decreased IGFBP1
  - increased IGFBP5.
